# To be or not to be co-infected

**DOI:** 10.1186/1756-3305-7-S1-O15

**Published:** 2014-04-01

**Authors:** S Moutailler, L Michelet, J Chotte, F Féménia, E Le Naour, M Cote, ML Poulle, E Vaumourin, P Gasqui, G Vourc'h, JF Cosson, D Raoult, M Vayssier-Taussat

**Affiliations:** 1 UMR Bipar, Enva, Anses, USC INRA, Maisons-Alfort, France; 2 URCA, SFR Cap Santé-PROTAL EA3800 et CERFE, France; 3 UR 346 Epidémiologie Animale, INRA, Saint-Genès Champanelle, France; 4 CBGP, Campus International de Baillarguet, Montpellier, France; 5 URMITE, Université de Médecine Aix-Marseille, Marseille, France

## 

Ticks can transmit a large spectrum of pathogens including bacteria, viruses and parasites with a significant number of these pathogens being agents of emerging infectious diseases. In Europe, the most prevalent tick-borne disease is Lyme Borreliosis, caused by the bacteria *Borrelia burgdorferi *s.l. In most cases, Lyme Borreliosis is well diagnosed. However, beside these typical cases, patients bitten by ticks can be infected by many other pathogens (bacteria: *Anaplasma *spp., *Bartonella *spp., *Rickettsia *spp.; parasites: *Babesia *spp., *Theileria *spp.; and arboviruses: TBEV) that are more difficult to diagnose. Moreover, co-infections between several of these pathogens might also occur. Clinical surveys show that patients coinfected by several tick-borne pathogens present more severe symptoms and a longer duration of illness than those infected by a single pathogen. The overall objective of our study was to evaluate tick-borne pathogen coinfection in ticks and the consequence of those coinfections for human health.

Using the high throughput real-time PCR chip, we detected the DNA of the 37 major tick-borne pathogens in a cohort of questing adult of *Ixodes ricinus *ticks collected in Ardennes (France). We identified that 60% of ticks were infected by at least one pathogen and half of the infected ticks were coinfected. We then evaluated the risk for simultaneous infection of those pathogens to humans by detecting the DNA of the most prevalent tick-borne pathogens in the blood of patients bitten by ticks. Our data illustrated the importance of coinfection, and highlighted the necessity to evaluate coinfection in the context of tick-borne diseases.

